# Actors, legitimacy, and governance challenges facing negative emissions and solar geoengineering technologies

**DOI:** 10.1080/09644016.2023.2210464

**Published:** 2023-05-16

**Authors:** Benjamin K. Sovacool, Chad M. Baum, Roberto Cantoni, Sean Low

**Affiliations:** a Center for Energy Technologies, Department of Business Development and Technology, Aarhus University, Aarhus, Denmark; b Science Policy Research Unit (SPRU), University of Sussex Business School, Brighton-Falmer, UK; c Department of Earth and Environment, Boston University, Boston, MA, USA; d Dipartimento di Scienze Sociali, Università di Napoli “Federico II”, Naples, Italy

**Keywords:** Carbon dioxide removal, greenhouse gas removal, solar radiation management, climate justice

## Abstract

Institutional theory, behavioral science, sociology and even political science all emphasize the importance of actors in achieving social change. Despite this salience, the actors involved in researching, promoting, or deploying negative emissions and solar geoengineering technologies remain underexplored within the literature. In this study, based on a rigorous sample of semi-structured expert interviews (*N* = 125), we empirically explore the types of actors and groups associated with both negative emissions and solar geoengineering research and deployment. We investigate emergent knowledge networks and patterns of involvement across space and scale. We examine actors in terms of their support of, opposition to, or ambiguity regarding both types of climate interventions. We reveal incipient and perhaps unforeseen collections of actors; determine which sorts of actors are associated with different technology pathways to comprehend the locations of actor groups and potential patterns of elitism; and assess relative degrees of social acceptance, legitimacy, and governance.

## 1. Introduction

Despite a long history of climate agreements since the Rio Earth Summit in 1992, greenhouse gas emissions have continued to grow over time, only temporarily slowed down by the first year of the COVID-19 pandemic in 2020. The expansion of renewable energy sources, energy efficiency measures, and the modification of production and consumption patterns have been amongst the more widely accepted strategies to address this upward trend. At the same time, a pair of varying established and potentially immature (or even hypothetical) socio-technological strategies have been proposed to address and ameliorate climate change: negative emissions technologies (referred to as ‘carbon dioxide removal’ or CDR) and solar geoengineering (also known as ‘solar radiation management’ or SRM). Whereas the more widely accepted strategies broadly entail actions that reduce how much greenhouse gas is emitted, such as shifting the sources of energy supply and demand or reducing deforestation and negative impacts of land use change, the novel ways in which these two new sets of strategies interact with and are impacted by the rest of the earth system – not to mention differences even within the sets of strategies themselves – motivates a more thorough consideration of the requirements, challenges, and benefits which they present.

But who are the actors behind such technologies? In this study, based on a sample of semi-structured expert interviews (*N* = 125), we explore the types of actors and groups of actors associated with both negative emissions and solar geoengineering research and deployment. We investigate emergent knowledge networks and patterns of involvement across space (including Global North vs. Global South actors) and scale (from global to local, large to small, and from individual actors to larger actor groups), thus providing a ‘geospatially thick’ description of our networks. Mapping them is crucial, especially considering criticisms surrounding elitism and colonialism in knowledge production and deployment (Winickoff *et al*. 2015, Biermann and Möller 2019). We lastly examine the stance taken by these actors in terms of their support, opposition, or ambiguity to both types of climate interventions.

Our assessment enables us to identify incipient and perhaps unforeseen constellations of actors; to determine which sorts of actors are associated with different technology pathways (e.g. negative emissions is envisioned as industry-led but solar geoengineering is government-led); to comprehend the locations of actor groups and potential patterns of elitism; and to assess relative degrees of social acceptance, legitimacy, and support across these actor groups. Although primarily empirical in nature, our study does advance recent debates occurring in environmental politics. Writing in this journal, Stephens *et al*. (2021) caution that deployment of radical climate interventions such as geoengineering could have grave political implications. More recently, Stephens *et al*. (2022) argue in favor of an effective international governance regime behind solar geoengineering technologies, and call for the involvement of the public. This call necessitates expanding the scope of discussion beyond just researchers-one assisted by our examination of all the non-research-related actors involved in both carbon removal and geoengineering. Mobilizing the advocacy coalition framework, Felgenhauer *et al*. (2022) argue that geoengineering currently lacks a strong advocacy coalition in the United States, given that actors are too weak or fragmented. Our study can reveal which emergent actors and coalitions are *behind* the politics, and particular areas of fragmentation as well as consensus.

## 2. Key concepts: actors, legitimacy, and governance

Institutional theory, behavioral science, sociology and political science all emphasize the importance of actors or groups of actors in achieving social change. Even though such multifarious actors and groups of actors can shape transition processes, policy change, or patterns of consumption via their material, social, and cultural agency, the institutional dimensions of low-carbon energy transitions remain underexplored within the CDR and SRM literature.

An ‘actor’ refers to an individual, or sometimes an entity, that makes a difference or effects the actions or outcomes of others (Latour 2005). In sociology and economics, an ‘actor’ can refer both to an individual, or a collective unit such as institutes and organizations, that engages in intentional action. In science and technology studies, a ‘relevant social group’ is used to denote the organizations that share the same set of meanings attached to a particular technology (Pinch and Bijker 1984, Bijker 1995). Social groups that constitute parts of the ‘environment’ for a technology play a critical role in shaping and defining the problems that arise during the development (and deployment) of an artifact; social groups thus give meaning to technology, and define the problems facing that technology.

Another important theme in our paper is that of legitimacy. We employ Schnedler and Vadovic’s (2011) definition of legitimacy as ‘a property of an institution, norm, or authority, [whose] key aspect is the process of validation, that is, an agreement among the members of the society that the course of action or type of behavior is in line with their moral values and principles of justice’. As we will see in Sections 4 and 5, legitimacy possesses multiple meanings and dimensions, with our study examining the legitimacy of actors, the legitimacy of actors’ potential decisions to deploy CDR and SRM technologies, and the legitimacy of the technologies themselves.

When actors reach consensus about the use of a technology, and have established legitimacy, closure can be said to occur. Closure and stabilization occur in technology when a consensus emerges among actors that problems arising in the development of technology have been alleviated, and/or an agreement emerges concerning a dominant frame among relevant social groups (Beder 1991). Closure can also reveal moments of leveraging the future, of ‘opening up’ narratives so opportunities for the ‘the orchestration of opportunity’ can be identified (Brown *et al*. 2000).

The final concept we employ in this study is ‘governance,’ which also connects to actor types. Drawing from Florini and Sovacool (2009), governance refers to ‘any of the myriad processes through which a group of people set and enforce the rules needed to enable that group to achieve desired outcomes.’ Extending from this literature on governance, our study investigates actor groups that perform governance across governments, intergovernmental bodies, private sector firms, civil society groups, members of research institutions, and the media.

## 3. Research focus, methods and limitations

With our key concepts briefly elucidated, this section moves to explain our selection of carbon removal and solar geoengineering technologies, our research design (including sampling, data collection, analysis, coding, and visualization) as well as limitations with our approach.

### 3.1 Focus on multiple technologies

The GENIE project focuses on the 20 distinct options or technologies shown in Table 1. This approach contrasts to other papers that were narrower in focus, i.e. only on carbon removal (Low *et al*. 2022a) or one individual technology like direct air capture (Sovacool *et al*. 2022) or space-based geoengineering (Baum *et al*. 2022). Research focusing simultaneously on both negative emissions and solar geoengineering can be contested (Jinnah *et al*. 2021).

**Table 1.** The 20 negative emissions and solar geoengineering options examined in this study.

Negative emissions and carbon removal	Solar radiation management and geoengineering
Afforestation and reforestation	Stratospheric aerosol injection
Soil carbon sequestration	Marine cloud brightening
Biochar	Cirrus cloud thinning
Bioenergy with carbon capture and storage (BECCS)	Space-based (extra-terrestrial) reflectors
Enhanced weathering	Albedo modification via human settlements
Ocean alkalinization or fertilization	Albedo management via grasslands and crops
Blue carbon and seagrass	Albedo management via deserts
Ecosystem restoration	Albedo management via clouds
Direct air capture and storage (DACCS)	Ice protection
Carbon capture utilization and storage (CCUS)	High altitude sunshades.

Nevertheless, there is a strong case to be made for looking at them comprehensively. First, they can work together as part of an effective climate intervention strategy. Delina (2021) argues that where carbon removal can be used to abate hard-to-mitigate sectors such as aviation or industry, lowering emissions, SRM can help reduce temperatures, enabling the international community to meet a temperature target of 1.5 to 2°C warming. Delina (2021, pp. 1–2) argues that ‘although the opinion on SRM approaches has been divided, both SRM techniques and CDR methods have been suggested to be crucial complements to climate mitigation via emissions reduction for meeting the goals of the Paris Agreement.’

Second, because both suites of carbon removal and solar geoengineering technologies occupy the same post-Paris space of incoming climate governance, our approach of investigating both sets of options has strong relevance to policy recommendations – it mirrors the policymaking dilemma of choosing options with limited resources and uncertainty, and advances understanding of how CDR and SRM options might work together as portfolio strategies or pose irreconcilable trade-offs (Sovacool *et al*. 2023).

Third, the ‘matching principle’ in environmental law (Butler and Macey 1996) states the scale of a solution ought to match the scope of the problem. In the case of climate change, that problem involves multiple countries, technological options, national pathways, policy mechanisms, and funding streams. One can extend the matching principle to infer that proper assessment must cover all relevant technologies shaping the political system concerning climate change. As such, a dual focus on CDR and SRM is warranted because they are exerting influence over climate politics. Including both therefore offers a more complete picture of politics, especially for understanding political lobbying across both types of technologies, the positionality of actors, and potential coalitions of advocates and opponents of both sets of technologies.

Fourth, a joint consideration on CDR and SRM is reflective of how our data was collected. In our interviews, we asked our expert respondents to discuss actors for both technologies. Limiting the discussion to only one of them artificially constrains the data and moreover omits some of the context of the data collection process. If nothing else, the fact that the separate discussion of carbon removal and solar geoengineering was one that was not universally shared by our expert participants, doing so could tend to be misleading, that is, of how our sample of experts viewed this issue.

Fifth and lastly, excluding one set of options (e.g. SRM) from analyses could drive discussion underground, beyond open and contestable research, even threatening to make it taboo. A resilient argument holds that excluding SRM approaches from scientific debate may not create a lasting deterrent, and it runs the risk that only the least responsible governments or groups, or those with the weakest safety standards or governance ideals, control the fate of the technology (e.g. Victor 2009). Others counter that given the history of global climate and energy politics, SRM (especially approaches with planetary scope) presents further incentives to delay decarbonization, or entrenches antagonistic geopolitics – both of which warrant a much more precautionary approach to further SRM assessment and funding (Stephens *et al*. 2021, Biermann *et al*. 2022). This debate continues, and we do not resolve it here. For now, we are hopeful regarding the benefits of encouraging stakeholders of all stripes and colors to continue to discuss CDR and SRM, fleshing out their risks, and debating when, if ever, initiatives should be deployed. This type of an approach has been utilized with other types of potentially hazardous materials and practices such as genetic research (the Human Genome Project), standards for genetically modified organisms (World Health Organization), nuclear weapons and power (the Nuclear Non-Proliferation Treaty), and the continuing study of dangerous diseases such as smallpox (International Task Force for Disease Eradication and the World Trade Organization). All of these efforts encouraged ‘responsible use’, and open scientific debate, rather than prohibiting use or silencing discussion.

### 3.2 Sampling, data collection and coding

Our research design centered on original data collected from 125 semi-structured research interviews with established experts, defined as those with comprehensive and authoritative knowledge about negative emissions and/or solar geoengineering research or commercialization, over the course of May to August 2021. We foreground that our experts were often speaking about immature or even hypothetical socio-technical systems – the data provided in this study are explorative mappings that represent propositional knowledge (Aven 2016) or claims with an eye to prospective inequities and other thematic concerns (Walker 2011). More details of our research design are offered in Annex I.

Table 2 shows an overview of the demographics of our sample, and Annex II lists all 125 experts who participated. We must note that despite efforts towards a diverse sampling, our focus on peer-reviewed publications had a particular screening effect, especially likely to have reduced our sampling of voices from NGO or activist groups, and our sampling of voices from the Global South. Our sample has a particularly strong bias for university experts. Although we did secure some interviews with members of civil society and nongovernmental organizations, governments, and commercial entities in the private sector, the sample is still strongly concentrated towards experts at universities and research institutes, which in a certain sense also reflects the composition of those currently working on these topics. That said, the sample does include scholars from more than 30 disciplines as well as a dozen participants from the Global South, here determined by either the country of origin of the participant and/or their current location. Given that interviewees were speaking on their own behalf, and given the sensitivity of the topic, the data from these interviews is presented here as anonymous with a generic respondent number (e.g. R10 for respondent 10, or R110 for respondent 110). All experts were speaking only in their personal capacity, not in an organizational capacity.

**Table 2.** Summary of the demographics of experts who took part in our study.

Summary information	No.
No. of experts	125
No. of organizations represented	104
No. of countries where experts are based	21
No. of academic disciplines represented	34
Cumulative years spent in the geoengineering industry or research community	881
Average years spent in the geoengineering industry or research community	7.8
No. of experts whose current position falls into the following areas:	
Civil society and nongovernmental organizations	12
Government and intergovernmental organizations	8
Private sector and industrial associations	12
Universities and research institutes	94
No. of experts from the Global South*	12

Note: The sum equals 126 due to the dual affiliation of one of our experts. *We categorized respondents from the Global South based on the classification provided by the World Population Review (2022). Many of these participants were selected from the DEGREES Initiative (formerly the Solar Radiation Management Governance Initiative), which engages with Global South countries and experts.

The full set of questions and sub-questions can be found in Annex III. Of specific relevance to this paper, we explicitly asked our experts: ‘Who are the relevant or most important actors (or stakeholders/networks) for the commercialization, development, and/or acceptability of these greenhouse gas removal and/or solar radiation management technologies?’ (Question 7 of 7). Of their own accord, our experts spoke about ‘relevant’ actors without us prompting them about what ‘relevance’’ means, to avoid potentially biasing results; respondents were empowered to define ‘relevant’ based on their own expert judgment. Our experts thus varyingly characterized relevance in terms of current (who matters now), emerging (who will matter soon), prospective (who could matter in the future), and normative (who they want to matter in the future) dimensions. Informants were also asked only about their self-declared areas of expertise – meaning many did not discuss all techniques, and some focused only on one (e.g. biochar, or direct air capture, or stratospheric aerosol injection).

Further details about our data analysis and coding are also offered in Annex I.

### 3.3 Data visualization

Finally, we visualized our actor data to make it more compelling. The number of interviews in which each actor was mentioned at least once – as well as each interview’s positioning of actors with respect to negative emissions, solar geoengineering, or both – were used to parametrize our network analysis through Gephi® software, and design the maps discussed in the results section. The thickness of the inter-actor links in the network maps indicates the number of times an actor was found supporting a specific positioning. An actor’s node size is proportional to the number of interviews in which that actor was mentioned.

For each technology, we proceeded as follows: starting from the file containing the actor names, the group each of them belongs to (if any), actor stances, and their frequency in the interview database (Table 3), we then created a three-column table only including binary ‘interactions’ between these three columns in the first two columns, and the frequency of each interaction (the interaction’s weight) in the third column. For example, from the original file we knew that for CDR, the ‘ENGOs and civil society group’ (predominant position: ambiguous) was mentioned 37 times, and included 10 actors, each with its own attitude. In the three-column table, we created binary correspondences between: ENGOs and ‘ambiguous’ (weight: 37); ENGOs and each of the ten mentioned NGOs; each NGOs and its attitude (with their respective weights). We proceeded in that fashion for all actors and groups. We then fed Gephi® the three-column table, adapting them in a format that was acceptable by Gephi®.

**Table 3.** An example from our actors and groups coding table.

ENGOs and civil society (37) (*ambiguous*)
a. Bellona Foundation (1) (*support*)
b. Friends of the Earth (1) (*ambiguous*)
c. Carbon180 (2) (*support*)
d. Carbon Removal Advocacy Europe (CRAE) (1) (*support*)
e. Greenpeace (2) (*ambiguous*)
f. The Green Alliance (1) (*ambiguous*)
g. Climate Action Network (CAN) (1) (*support*)
h. Carbon Market Watch (1) (*support*)
i. “White-shoe NGOs” (…) (1) (*support*)
j. Right-wing actors/“Querdenker” (2) (*support*)

After importing this data into Gephi®, we chose appropriate parameters and layout for the final visualization, manually adjusting the graphics so that it the diagram could be as easily readable as possible. Due to the number of actors involved, however, the final graph appears quite crowded. Thicker lines in the graph indicate the weight of support, opposition, or ambiguousness. Node size is determined by degree centrality, which measures the number of incoming and outgoing relationships from a node (i.e. the number of occurrences of single nodes). The thickness of interconnecting lines is proportional to the frequency of co-occurrences of two specific nodes in the database. While this diagram has a quantitative base (e.g. ‘Scientists’ expressed ambiguousness about three times as frequently as ‘Public and society’), we chose to represent it qualitatively.

### 3.4 Limitations

Although the large number of experts interviewed does have its strengths in terms of qualitative rigor and the scope and diversity of perceptions reflected, our approach does have some limitations. In our study, we interviewed experts about who they deemed to be the most influential actors or groups in the space at present, rather than interviewing the actors themselves. This was done, *inter alia*, because we needed in our research to first identify and map *who* the most relevant actors were, at present, for the development and (at times) deployment of the various climate interventions, thereby relying on the expertise of our participants to do so. A logical next step would therefore be, on the basis of these insights, to then undertake empirical research approaching all of the actors identified. As a result, the data and results should not be taken to reflect ‘these are the most important actors’, but rather that ‘these are the actors who experts perceive as most important.’

Moreover, one drawback to the anonymity of our respondents is that there is no guarantee this study can be replicated, because the authors cannot correlate the identity of respondents with interviewee statements.

Furthermore, our interviews were more empirically orientated than conceptually grounded, which is why we refrain from advancing any single environmental politics theory or framework in this study.

Finally, we took an ethnographic approach that did not correct or problematize responses, so we present the data unfiltered, even if our respondents may have had misperceptions about the actors involved in CDR and SRM. In the end, we determined that, rather than impose our own views on those of experts, i.e. to ascribe validity or reasonableness to their statements, the best approach was to defer to the experts themselves.

## 4. Results: actor types, legitimacy, and location among actor groups

This section organizes our results across three core themes: the coalitions and types of actors across different technologies; social acceptance aspects of their stance for or against the technology; and spatial aspects of their location and scale.

### 4.1 Groups: types of actors and relevant social groups

Our data points to very different relevant social groups emerging for negative emissions technologies contrasted with solar geoengineering technologies. As R056 surmised:

*It’s almost opposite [actors involved] for the two different types of interventions. On solar geoengineering, it still is to this day basically a group of a few dozen or maybe a hundred scientists, pragmatic environmentalists, philanthropists, tech visionaries, and of course the opponents. You know, the anti-technology environmental groups. On carbon dioxide removal, it’s almost the opposite situation. You have the leading environmental groups that have enthusiastically bought into nature-based solutions, the policymakers and legislators who have breathed a sigh of relief at the maneuvering space that comes from that change of rhetoric and have started pouring gazillions of dollars into research support, and the explosion of sort of small commercial life that’s come up with dozens and hundreds of firms.*

R047 identified divergent funding and actor networks involved with each set of options as well, stating that:

*The value of innovation is different for negative emissions compared to solar geoengineering. The value in SRM and innovation spending is a public good […] there is no real role for private sector other than maintenance, lift services from a SpaceX or Boeing, private vendors but on a contract for service … For carbon dioxide removal [or negative emissions], it’s a very different world, a blend of public and private goods, private good stuff will take off, look at the current mania around net zero. It has spun up the accounting department, and not the innovation department, it’s all about maximum volumes and minimum costs, [and it] shows you private sector business model is powerful, but to get that one right, [we] need early stage deployment.*

This statement implies a limited contract for service model for solar geoengineering but a much broader and more valuable global market for negative emissions.

**Figure 1.**
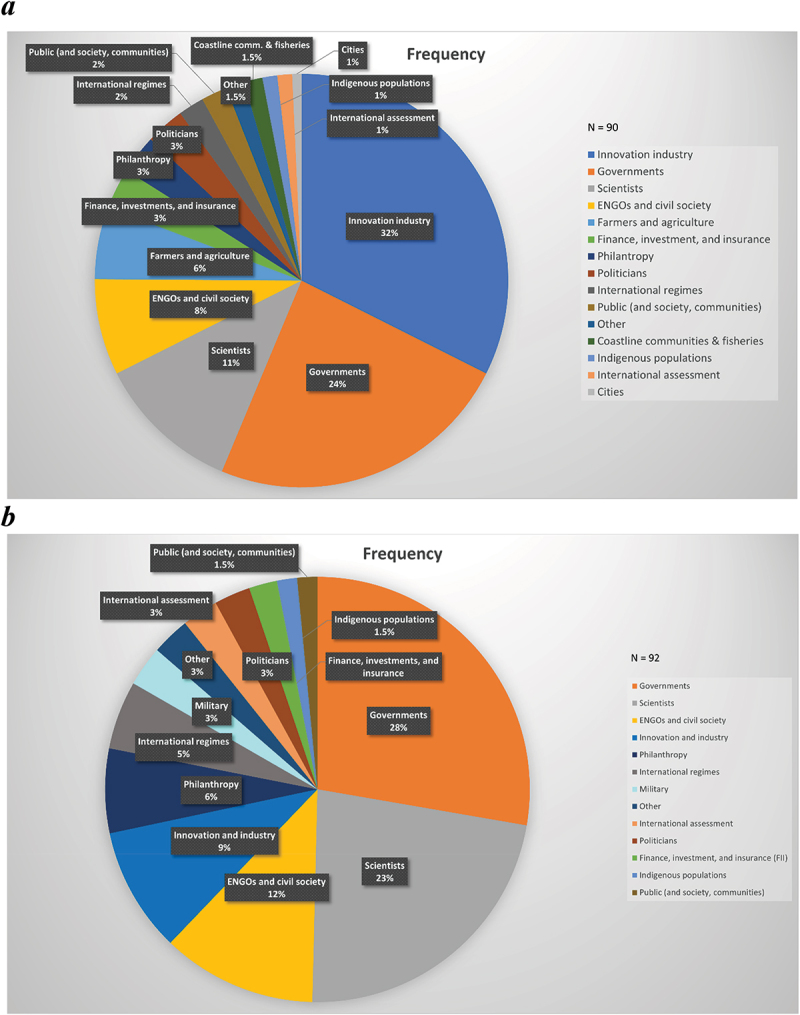
Relevant social groups for negative emissions (Panel A) and solar geoengineering technologies (Panel B).

Note: based on expert research interviews (*N*=125) who were asked to determine “the relevant or most important actors for the commercialization, development, and/or acceptability of greenhouse gas removal and/or solar radiation management.” The diagram depicts the frequency of occurrence across the entire interview sample. The category of “governments” refers to those within actual ministries, so-called “bureaucrats,” whereas “politicians” refers to those within a particular political party.

Indeed, across all 125 of our interviews, Figure 1 does depict different relevant social groups with each type of technology. The most relevant negative emissions actors are led by industry, followed by governments and scientists; there is little mentioning of the involvement of cities or the public. R064 explained the industry and government attraction to negative emissions as follows:

*In the case of carbon removal, the most important actors will be national or subnational policymakers, large landowners, and/or people who control land use.*

*I think there are going to be a lot of big, commercial actors in this space. It’s expensive to do at a large scale, which means someone, or some group of people will be making a lot of money, presumably, and so that brings in all kinds of political economy implications.*

The implication is that technology deployment is driven by large-scale policy incentives and big commercial actors.

Cities, by contrast, are not viewed as a currently important actor, nor are civil society groups. R024 argued that cities and urban areas ‘*don’t know yet they are an important actor, they are mostly thinking about urban greens and urban plants, cities don’t see it as carbon dioxide removal, or albedo modifications, or buildings as a carbon stock, or cities as a carbon sink*.’ R027 also cautioned that civil society groups are not yet widely involved in either sector, even though they should be, given that they can ‘*perform a really important role of reminding us of the risks of technological hubris and the risks of moral hazard* …’ R045 also elaborated on this theme of the noninvolvement of civil society and local political actors in either suite, noting that they remain almost non-actors or invisible actors. As they explained:

*What is interesting in the absence of certain actors: the involvement of only a small number of some think tanks, who have only really dabbled in it, not really any serious players. Conservatives and politicians are not really taking an interest. I expected them to become substantial players, but they haven’t. Political parties have been virtually absent in the discussion so far.*

Panel B of Figure 1 reveals slightly different actor groups and dynamics behind solar geoengineering. The most relevant actors identified by our respondents here were governments (prospectively or normatively), followed by scientists and civil society – although as we will explore more in Section 3.2, relevant does not necessarily mean powerful. Similar to carbon removal, we see less involvement of industry, and again marginal involvement of the public or indigenous peoples. R009 spoke about within this fragmented collection of actors, ‘*the most important community are the intermediaries, the negotiators, the enablers, the people who can move beyond all of these actors and create a platform for having these societal debates, and unpacking the issues*.’

One core finding arising from Figure 1 is that no actor type is thought to dominate current discussions and debates about who is relevant to negative emissions or solar geoengineering – though some actor types command strong pluralities. Our respondents affirmed this point as well, with R045 stating that ‘I don’t view any single actor as powerful, and the existing power of actors is being diluted as more people come into the debate.’ R081 believed that ‘only a few academics and a few industrialists who think they can make money’ are involved, with ‘no policymaker seriously talking about it.’

Still, support for negative emissions as a broad strategy is growing rapidly in the context of Net Zero commitments, and given its inclusion in the most ambitious mitigation pathways of the Fifth and Sixth Assessment Reports from the IPCC. We might expect governmental actors (for providing incentives and regulation), as well as innovation and industry, to maintain leading roles in upscaling negative emissions. Still, deep uncertainties remain on the risks and co-benefits of diverse, often-immature approaches in different geographies – if the topic becomes more central to climate policy, a host of on-the-ground stakeholders and publics will come into play. Solar geoengineering currently remains the province of scientific assessment – but the pressing implications for (climate) security may spur the concerted input of (inter)governmental actors and forums.

### 4.2 Legitimacy: acceptance or contestation in actor stances

**Figure 2.**
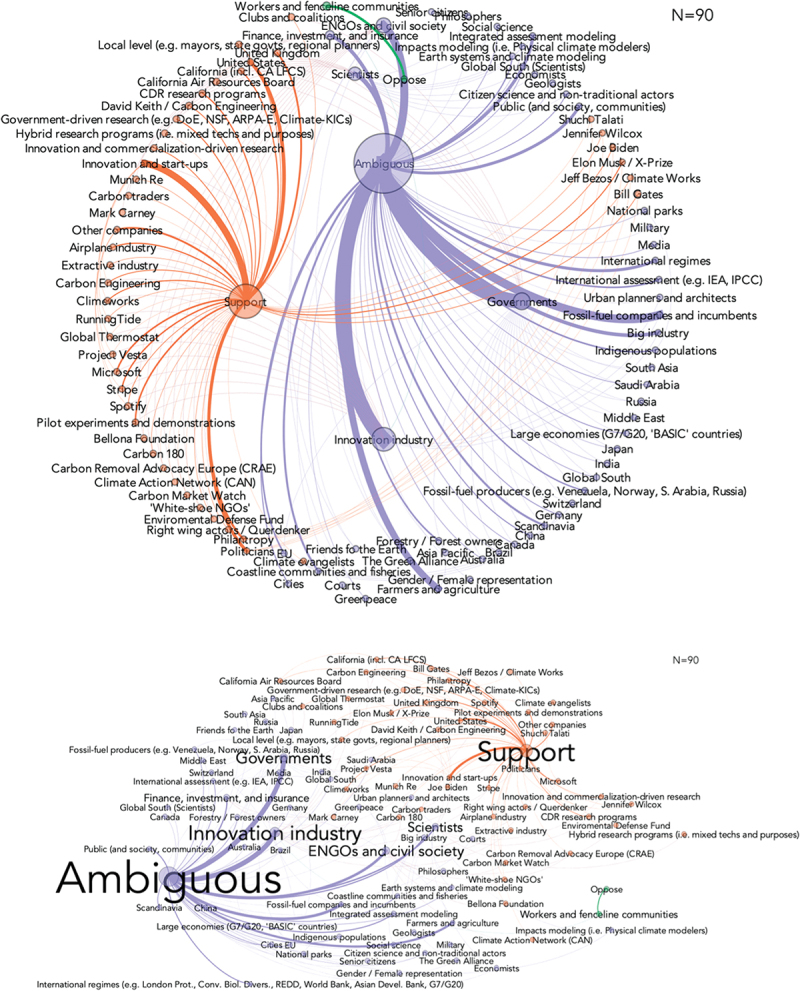
Actor stances and network positionality for negative emissions technologies.

Note: based on expert research interviews (*N*=125) who were asked to determine “the relevant or most important actors for the commercialization, development, and/or acceptability of greenhouse gas removal and/or solar radiation management.” The diagram depicts stances across the CDR-relevant interview sample (*N*=90). Orange indicates support, purple indicates ambiguousness, and green indicates opposition. Support may mean the actor supports research but not necessarily deployment – and that how these activities are perceived might differ across expert participants.

**Figure 3.**
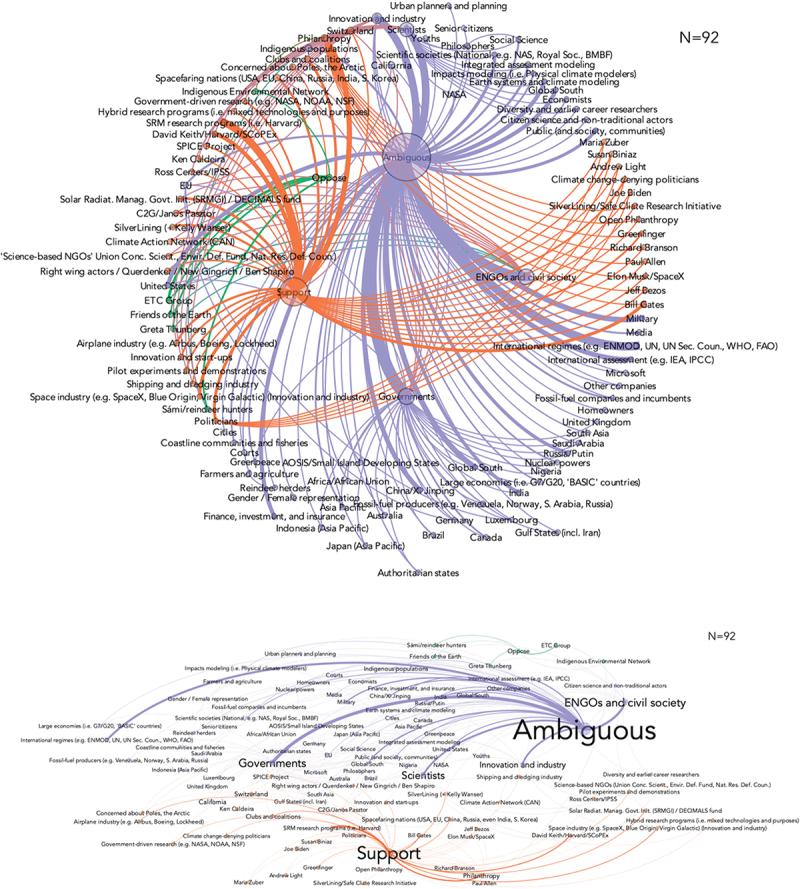
Actor stances and network positionality for solar geoengineering technologies.

Note: based on expert research interviews (*N*=125) who were asked to determine “the relevant or most important actors for the commercialization, development, and/or acceptability of greenhouse gas removal and/or solar radiation management.” The diagram depicts stances across the SRM-relevant interview sample (*N*=92). Orange indicates support, purple indicates ambiguousness, and green indicates opposition. Support may mean the actor supports research but not necessarily deployment – and that how these activities are perceived might differ across expert participants.

Actors have a particular stance that they publicly take in terms of accepting and supporting either negative emissions or solar geoengineering; opposing and criticizing it; or adopting a more ambiguous or ambivalent position. Figure 2 maps actors by their determined stance (using the whole of research interviews for necessary context) for negative emissions technologies. It indicates a far broader array of actors taking ambiguous stances, followed by a cluster of actors in support and a small number (workers and fenceline communities, some civil society organizations) in opposition. Figure 3 maps stances for solar geoengineering options, with a similar large concentration of ambiguous actors followed by those in support with a slightly larger number of actors in opposition.

The prevalence of ambiguous actor stances underscores that the technologies, with the probable exception of nature-based approaches such as ecosystem restoration and afforestation and reforestation, broadly remain contested and also that they lack what is called ‘closure’ (see Section 2), being so new. Such contestation, however, is mostly intra-scientific. If they were more broadly contested by social groups, Figures 2 and 3 would reveal far more oppose-support polarization, whereas our analysis reveals ambiguousness and some support, and close to no opposition. The implication is perhaps that these technologies have not *yet* had the chance to get contested widely, as they are relatively new and – especially solar geoengineering – have not yet been implemented. The few actors that might oppose them are thought to be those that might suffer from proximity to pollutive or hazardous siting of facilities. As for solar geoengineering, the opponents’ pool looks larger and includes persons and environmental NGOs with strong positions on rapid decarbonization (e.g. Greta Thunberg and Friends of the Earth), and whose positions contest what they see as engineering or entrepreneurial hubris.

Another possible explanation for the degree of ambivalence within our data to both CDR and SRM, and the lack of opposition to even SRM, is that to our experts SRM may be viewed as desirable enough, or at least not undesirable enough to oppose outright. At least in the case of solar geoengineering, most if not all observers acknowledge problems and risks, but some ultimately say the benefits of further research outweigh the risks. Thus, even some of the experts we interviewed, or the actors they identified, who may support or be ambivalent about CDR and SRM, may still find them not to be wholly unproblematic.

Moreover, orchestration is nascent for negative emissions and solar geoengineering technologies, and existing support may be temporary. R005 expressed concern that ‘*these options could face a “kiss of death” if sustained social opposition occurs*.’ R026 noted that solar geoengineering options in particular are ‘*very controversial*’ and that deployment will meet ‘*serious objections from international NGOs; I worry it will always face extreme opposition*.’ Speaking to negative emissions, R085 noted ‘*deep and widespread opposition to the scale up of direct air capture if done today*,’ opposition that could be *‘dramatic and latent’* in a way that fooled policymakers into thinking they had social legitimacy for deployment until they were stopped by losing that legitimacy. R011 even termed this as a ‘sticky slope’ problem of deployment:

*Some options like stratospheric aerosol injection are downright scary and would require some frightening technologies to become a reality, things like the U.S. Navy using 16-inch guns to fire particles into the stratosphere or using drones to spray sulfur into clouds. The SCoPEx* [a scientific experiment to advance understanding of stratospheric aerosols that could be relevant to solar geoengineering. Authors’ Note] *people couldn’t even fly a single balloon in Sweden, this shows you how difficult it may be, it may be socially impossible to do this. Many opponents worry about a slippery slope to deployment. I think it will be the opposite: a sticky slope. The more we do some of these options, the harder it is socially and politically, preliminary deployment oddly reduces the probability of actually doing it.*

These statements all imply that deployment and upscaling, particularly for SRM and engineered approaches of CDR, will face unresolved controversy, a case already exhibited with analogous technologies perceived as ‘high risk’ such as nuclear power where serious mismatches between social expectations and actual experience with deployment occur.^31^

**Figure 4.**
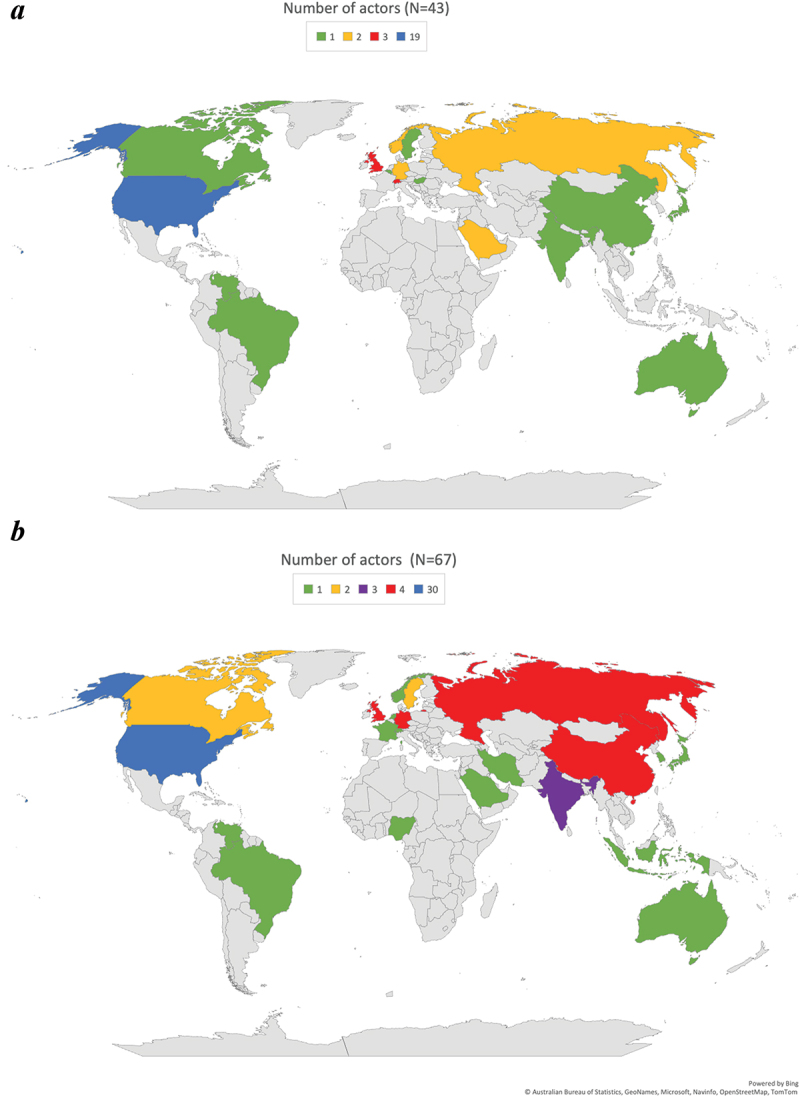
Mapping the location of relevant actors for negative emissions (Panel A) and solar geoengineering technologies (Panel B).

Note: based on expert research interviews (*N*=125) who were asked to determine “the relevant or most important actors for the commercialization, development, and/or acceptability of greenhouse gas removal and/or solar radiation management.” The diagram depicts the location of those actors. Mapping did not account for the frequency by which actors were mentioned, just their location. Global actors or institutions did not get classified, e.g. Greenpeace (which operates in 55 countries) or the Climate Action Network (1,300 NGOs across more than 130 countries), or the United Nations, nor were groups of countries (e.g. Small Island Developing States, or African Countries, or the Global South, or “authoritarian states”). General actors (farmers, evangelists, scientists, mayors, etc.) were also not classified in this part of the analysis. However, national institutions including civil society groups or universities did, i.e. Harvard University for the United States or Norway for the Bellona Foundation. The size of each of the two samples is different, as shown in each diagram’s N, as not all respondents mentioned both technology classes.

Additionally, the variation and lack of any single frame or accepted stance shown in Figures 3 and 4 reveal that no single actor currently dominates any network. In simpler terms, there is no concentration of power. R056 elaborated on this point as follows:

*No actor has power. It’s the same people again and again who have been frustrated about climate change but unable to really stop it. It’s a tiny world. Momentum has even sort of stalled internally by the effective opposition to research on options such as solar geoengineering. That’s probably going to break loose soon. I sort of want to say there’s no one powerful because there’s so little happening. Right? It’s like asking, you know, who are the big powers in the Bridge club at my mother’s church? There’s actually very little concentration of power in the sector at present, for both sets of options [negative emissions and solar geoengineering].*

Other respondents noted when considering our question about relevance that ‘relevant’ does not necessarily mean ‘powerful’ or ‘legitimate.’ R099 identified ‘*only a few meaningful actors*’ in each case and added that ‘*relevant doesn’t mean legitimate, it means actors that are taking decisions and moving the debate, but not necessarily the right actors*.’ R087 agreed when they remarked that ‘*I don’t think there is much power, people are trying to do things, but nobody has much power, in fact all I see are a bunch of powerless people who would like more influence*.’

### 4.3 Places: the location and scale of actors and groups

Actors and coalitions are lastly geographically located or embedded. Studies agree on a concentration of effort in the Global North, but differ on whether this reflects an unsalvageable inequity, or whether debate can usefully expand to the Global South. Biermann and Möller (2019) identify what they see as a pattern of intellectual colonialism in that knowledge production remains led by major research institutions (and the interests backing them) in Europe and North America, making it a ‘rich man’s solution’.

Knowledge and capabilities remain concentrated mostly in industrialized countries, perpetuating ‘hidden politics’ and inequalities in decision making authority and also shaping the types of research projects undertaken in the first place (Jinnah and Nicholson 2019). With specific regard to solar geoengineering, Stephens and Surprise (2020) cautioned that most research was being done by primarily white men at elite organizations in the Global North, with funding from billionaires. Táíwò and Talati (2022, p. 13) retort – with specific regard to solar geoengineering – that research in the Global South may be useful, and that those in the Global North should stop insensibly arguing ‘from the armchair about what could be beneficial for global justice and declare less on other people’s behalf.’

Figure 4 maps the locations of 110 actors, doing so specifically for 43 actors deemed relevant for negative emissions technologies and 67 for solar radiation management. As Figure 4 indicates, the United States remain the actor epicenter of both classes of technology, with strong accompanying concentrations in central and northern Europe. In solar geoengineering, this likely reflects that the most visible and vocal advocates for further research are concentrated in the US (National Academies of Sciences 2021), although there are efforts to investigate marine cloud brightening for protecting coral reefs (Australia). Negative emissions reflect a comparative varied and dispersed scene – leapfrogging academic assessment into innovation and industry, and increasingly into policy proposals and formation. Much of this also reflects a greater variety in kinds, components, and supply chains of novel carbon sinks – from biogenic (agricultural and forestry management, blue carbon) to engineered (direct air capture), to chemical approaches (enhanced weathering). Some countries are highlighting their storage capacities (Norway), some are interested in forestry feed-ins (Sweden), some are hosts to companies piloting small-scale direct air capture facilities (Carbon Engineering in Canada; Climeworks in Switzerland). Others may be aiming to be leaders in carbon capture and storage (the United Kingdom) or bioenergy with carbon capture and storage (Brazil).

The map may also indicate similarities in technical capabilities that cut across both negative emissions and solar radiation management – for example, locations with strong basic science research enterprises, environments with large amounts of public and private funding, and necessary engineering procurement and construction skills.^37^ The dominance of Global North countries in these networks, especially those in North America and Europe, has been interpreted by some of our respondents as elitist. R042 spoke about how such climate interventions are by their nature exclusive because ‘*billionaires, who have money to play with, do it because they can, other people cannot*.’ R045 similarly cautioned against an emergent ‘*geo-elite*’ of well-resourced individuals with techno-optimistic views driving investigation into controversial forms of climate action. As they stated: ‘*I could see a billionaire megalomaniac, kind of Dr. Strangelove, trying to deploy … those likely to have a kind of Messiah complex. Someone who thinks, I am going to save the world*.’ Hamilton discusses the dominance of science-policy networks in the Global North, insofar that political decision makers will become highly dependent on well-funded technocratic bodies in order to ensure such technologies function.^38^

A final aspect of space and scale arising from our material relates to governance, or the inadequacy of current governance arrangements to coordinate and manage the network visualized in Figure 2. For instance, R085 spoke about the need for far more democratic, participatory planning related to these options, emphasizing the need for ‘*broadly shared, inclusively developed*’ set of scenarios or shared assumptions for evidence-based dialogue. R085 argued that the current scenarios utilized by climate science are ‘*wildly inadequate*’ to the task. R064 also mentioned that ‘*the governance mechanisms out there are not up to the task, and it is unclear how to move forward*.’ R063 framed the governance challenge as ‘*unlike anything before*’ and stipulated that an entirely ‘*new multi-sectoral governance enterprise is needed*.’ As they went on to explain, regarding a fully-scaled negative emissions economy:

*For net-zero to work, we will need to build one of the largest sectors in the world, over multiple decades and even realistically centuries, with accurate flux and stock numbers, in perpetuity. We will need to develop a specific governance system, as we cannot retrofit and fumble through way through it. This is a – sort of – Wright brothers, ‘Man on the Moon’ challenge or transformation. This is a completely new construct, a new type of transition we have never done before … the institutional architecture will rival almost every national treasury on the planet … we are talking about 3–4 times the amount of oil and gas our entire system produces, but going in the other direction.*

This institutional architecture, meant to refer to the social support systems of finance, governance, and policy behind the technology, they concluded is ‘*nascent and non-existent*.’

## 5. Discussion: closure, networks, and future research directions

The actors and groups behind climate interventions such as negative emissions and solar geoengineering technologies are as varied as they are spatially diverse, with inchoate networks forming supporting, opposing, and mostly remaining ambiguous about future deployment. No closure yet exists where these technologies are stabilized, and relevant social groups continue to extract and expand as the technologies coevolve with policy regimes and social perceptions. Power is dispersed, actor networks shifting, legitimacy remains uncertain and contested.

In our interviews, some actors were mentioned more frequently than others as possibly relevant with respect to the deployment of negative emissions and/or solar geoengineering, but the ambiguity we found across groups of interests is also partly reflected in the unclear positioning of single actors within each group of interests. An exemplary case is the innovation and industry group, an umbrella sector that is generally ambiguous or supportive of negative emissions (an ongoing wave of corporate Net Zero commitments, innovation opportunities, and time-buying rationales are often cited), but where the added burden of future regulations might generate opposition in the future. With respect to solar geoengineering, a similar pattern is found in the ENGOs and civil society sector, which is generally ambiguous vis-à-vis these technologies, but includes positions as diverse as Friends of the Earth’s (opposing), Greenpeace’s (ambiguous), and right-wing actors’ (supporting) – as is evidenced in Figures 3 and 4. Hence, the categorization of ‘macro’ groups of interests is only an indication of the prevalent positionings within it. For both technologies – but more clearly for negative emissions than for solar geoengineering – the networks show strong connectivity between the generally ambiguous large groups of interests and their sub-nodes, namely individual actors, most of whom are either ambiguous or supportive. This may be an indication of the future orientation of the whole group of interest. Speculatively, ambiguous actors could be more likely to turn supportive than opposed if the group of interests is already partly oriented towards supporting.

Interestingly, for both negative emissions and solar geoengineering – but more for the latter than the former – the network of opponents is rather small. Contestation seems to be quite reduced in scale. However, rather than taking that as a proof that these technologies are unproblematic, the explanation could be that these, in some cases, have not *yet* had the chance to become widely contested, as they might be immature or even hypothetical, less publicized than other strategies to counter climate change (for example, the expansion of renewables), and – especially solar geoengineering – at the experimental stage. Or, that there are still enough ostensible benefits to each technology that justify giving them consideration, thus situating people in the category of ambivalent, rather than outright supporting or opposing them. Locally impacted communities may latently oppose negative-emissions projects and facilities. As for solar geoengineering, the pool of potential opponents looks larger and already includes vocal ENGOs and some local indigenous communities (Low *et al*. 2022b), not to mention how such a pool might grow should the drive towards early deployment come to be identified with relatively unknown start-ups (e.g. Temple 2022). Furthermore, the positioning of vocal ENGOs and local indigenous communities is possibly related with contestation of engineering or governance hubris (Owen 2014), moral hazard or mitigation deterrence (McLaren 2016) – namely, the danger that policymakers may reduce mitigation efforts by turning to strategies that would ostensibly buy time – or the threat that pilot projects are tested or implemented in areas where indigenous groups conduct their daily activities.

There is timeliness to insights on emerging actor networks as well, a temporal dynamic that shapes current and future patterns of legitimacy and social acceptance. Some actors – governments attempting to incubate research and development, leading research programs, or global assessment bodies such as the IPCC, thought entrepreneurs, a burgeoning innovation space for early-stage carbon removal technologies, or key oppositional ENGOs – clearly matter at the nascent stages of technology, i.e. ‘now’ or between 2022 and 2030. Other actors, namely, most governments determining their climate commitments, civil society groups active in climate spaces, national and regional publics, and groups of financiers are not immediately involved. But they will come to matter in the mid-term (e.g. between 2030 and 2050) as debate over the place of negative emissions approaches and solar geoengineering in climate action grows.

Still other actors – or configurations thereof – are prospective. For solar geoengineering, mini-lateral ‘climate clubs’, or non-climate global governance regimes (e.g. the United Nations Security Council), could become important, along with national militaries (for solar geoengineering and weather modification techniques). The delivery of stratospheric aerosol injection in particular could produce a strong subeconomy for engineering and aerospace development. For negative emissions, political leaders, or locations with large assets (land, resources and technological capabilities) could matter in the future, as more learning occurs and deployment scales up. Prospectively important actors include agrarian polities and communities where farming or ecosystems services are critical to the economy; conservation groups and national parks for forestry management; and marine communities, fisheries, shipping, dredging, and coastal economies for enhanced weathering or marine-based negative emissions approaches. Fossil fuel producers (industries and national economies) are prospectively important, given their capacity for powering direct air carbon capture and storage or sequestering carbon. The co-benefits of different types of negative emissions approaches could also prospectively benefit different sectors (e.g. cement, plastics, fertilizers, etc.). Finally, others spoke about more normative aspects of who *should* matter more, namely research communities in the Global South or potentially vulnerable communities hosting projects.

Finally, our empirical results can help inform a future research agenda concerning the actors of CDR and SRM that is more theoretically and conceptually grounded. In their survey of theories of policy relevant for energy and climate transitions, Kern (2018) identify five different approaches, the discussion of which our results can inform. The central idea embedded within the *advocacy coalitions framework* is that actors that regularly participate in policy formation shape a policy subsystem through their coalitions, who compete for influence. Whereas our results have begun to distinguish areas of consensus and dissensus, future work could examine the core beliefs that actors hold, as well as their attempts at coordination and the stability or fragility of the coalitions they form. A fuller mapping of actor coalitions would reveal the sorts of activities CDR and SRM actors are generally engaged, that is, in beyond research, deployment, or acceptance in the broad sense of supporting vis-à-vis opposing. A key component of the *multiple streams approach* is that actor behavior, especially misbehavior, can be driven by non-rational values and beliefs; it also advances the notion of policy entrepreneurs, that is, overly influential actors who are able to determine the agenda-setting process. Future work could build on our results to identify which actors may become policy entrepreneurs, and which actors may rely more on non-rational forms of policy persuasion – not to mention, the kinds of actors that take on particular importance for public outreach and communication. *Punctuated equilibrium theory* examines how actors define problems and set agendas in the policy process but are stymied by overlapping jurisdictions, changing resources, and shifting political alliances, all of which result in disruptive and unpredictable cycles of continuity and change. Future work building on our results could thus explore which actors are effective at setting agendas, and which ones are able to harness incremental change vs. more radical contestations and shifts in policy (i.e. punctuating an existing equilibrium). Actors can rally around not only technologies but *discourse coalitions*, where they come to share common assumptions of reality, usually enacted through narratives and visions. Future work could build on the present study to explore which narratives, visions, and discourse are being employed by various actors and actor groups. Finally, *policy feedback theory* suggests that climate or energy policy is not merely a product of politics, but actively shapes future policymaking opportunities itself. Actors mobilize into networks that come to create self-reinforcing, positive feedback processes known as path dependency. Path dependence is crucial to identify and consider, given how its existence can limit the viability of certain policies or actions and to generally limit the scope and space which actors can have to maneuver and take decisions (Mahoney and Schensul 2006, Torfing 2009). And so future work could explore the degree to which actors are able to create, or challenge, the path dependency of any of our 20 CDR and SRM options.

## 6. Conclusion

Advocates and sponsors of negative emissions technologies may hope and desire that these systems are viewed only as forms of energy infrastructure that are safely storing carbon dioxide, whereas advocates of solar geoengineering may wish that it is seen only as climate protection infrastructure harnessing low-tech devices such as aircraft and balloons to stabilize temperature. Contrary to such analysis, the research conducted here of 125 expert interviews proposes the existence of far more varied views and perceptions when it comes to the actors involved, even for options that are already being implemented, such as various CDR options (notably afforestation, ecosystem restoration, soil sequestration, biochar, BECCS and to a degree DACCS), or those emerging in experiments, such as enhanced weathering, marine cloud brightening, and stratospheric aerosol injection. Assessments that ignore these (sometimes hidden) social dimensions threaten to naturalize them as part of the normal environment and depoliticize the contested stances of many actor groups.

Although no definitive actor or group dominates current deliberations about CDR and SRM, emergent networks could be simultaneously decentralized or dominated. New advocacy coalitions could form, placing them into the mainstream, further supported by agenda-setting processes or multiple streams that use non-rational values (such as fear or urgency around a climate emergency) to persuade planners to adopt and nurture them. Compelling new visions could emerge that lead to entirely new discourse coalitions, and the path dependency of policy processes could either cement CDR and SRM options into climate planning or prevent them from interfering with the existing path dependence of climate mitigation or adaptation.

Both negative emissions and solar geoengineering technologies contain what science and technology scholars would call ‘an interpretive flexibility’ given that various social groups (stakeholders) continue to attach different, and at times conflicting, meanings to them. Climate protection is, in other words, polysemic – it will provoke contrasting reactions based on both the type of relevant social group and the nature of the actor network. It may very well be that it is the interpretative meaning attached to them which will determine whether they blend into the global energy system in a seamless way or not, and as a result whether they will ultimately succeed or fail. In other words, it could be these actor dynamics, not hardnosed energy and climate policies by themselves, which will eventually decide whether deployment will occur at scale – or not.

## Supplementary Material

Supplemental Material
