# Supplementary material for: Actors, legitimacy, and governance challenges facing negative emissions and solar geoengineering technologies
Source: Env Polit. 2023 May 16;33(2):340–65. doi: 10.1080/09644016.2023.2210464 (PMC10911681; doi:10.1080/09644016.2023.2210464)
Supplement: Supplemental Material [file FENP_A_2210464_SM3182.docx]

# Annex I: Additional details of our research design

To ensure the credibility of our knowledge base, our recruitment and sampling of experts was triangulated from the following criteria. We aimed firstly for a diversity of stances (advocative or critical positionings), disciplines (technical and engineering sciences, economics, environmental and climate science, social sciences and humanities), sectors (research, technology development, government and policy, NGO), and expertise on technologies in question (‘climate geoengineering’ as an umbrella category, solar geoengineering or negative emissions suites, or individual approaches).

We further screened all potential participants for those who have published academic peer-reviewed research papers on the topic – again, with an eye to diversity in disciplinary or sectoral expertise as well as stance – or published patents and intellectual property, within the past ten years (from 2011-2020). This was meant as an (imperfect) proxy for expertise, as well as recent or ongoing involvement and commitment to the subject matter, and its assessment and governance. This criterion was applied even to participants outside of academia, e.g., from civil society or the private sector, to ensure a common baseline of expertise and knowledge.

In terms of data analysis and coding, our paper deals with expert responses for *all 20* negative emissions or carbon removal (CDR) and solar geoengineering (SRM) options being examined in the GENIE project, and we use the full sample of 125 interviews (whereas other papers in the project have used a subset of respondents). Although a plurality of experts discussed both sets of technological options, 33 focused only on CDR, 35 focused only on SRM; overall, 92 engaged with SRM when answering at least one of the questions, and 90 CDR.

Our broad approach across negative emissions and solar geoengineering technologies is matched to our data collection techniques: we asked respondents about all options. We did not force respondents to be either narrow or broad - we left the focus to them. Notably, many raised issues of splitting vs. lumping, and many also pointed out that the same risks or actors or venues emerge across different approaches.

The interviews were semi-structured, meaning that in every interview, all central questions were asked, but depending on answers, not all sub-questions were necessarily asked; these were asked only when relevant and only when they fit into the conversational flow of the interview. This semi-structured approach conforms to standard qualitative research methodology (Longhurst 2003; Newcomer et al. 2015). All interviews were recorded, fully transcribed, and then coded via NVivo. Analysis was thematic and inductive, with perceptions of actors, space, and scale coded and recoded iteratively within the research team.

Having had two members work iteratively and in parallel on coding all of the transcripts from the expert interviews in a tripartite fashion (i.e., first according to the relevant question, then according to the pertinent technology or technologies; and then in relation to the thematic content involved), interspersed with and buttressed by discussions and reconciliation over how statements were coded, the statements related to CDR and SRM were analyzed in the following procedure. First, the statements for each of the nodes and sub-nodes (e.g., ENGOs and civil society; governments; innovation and industry; and scientists) were segmented into separate blocks for SRM and CDR, with those referring to both allocated once each to each of the categories. Then, a numerical frequency count was done for each of the nodes and sub-nodes in terms of how many experts mentioned a particular actor group or sub-group, thus facilitating a comparison of, *inter alia*, how often each of the actors were mentioned for SRM vis-a-vis CDR as well as how often different types of groups (such as scientists versus the public versus innovation and industry) featured in expert discussions plus whether certain sub-groups of an actor group were more prominent, for instance, the United States versus Russia or Brazil under "governments" for CDR or the ETC Group versus "Science-based NGOs" under "ENGOs and civil society" for SRM. We highlight that this frequency count represents the number of experts which mentioned the actor group, rather than the number of statements made - in order to avoid repeated mentions by an expert overly coloring the final numbers. Lastly, in order to advance a summary judgment of the overall balance and tenor of expert statements, an assessment was made of whether those experts mentioning a particular actor group deemed their relationship to CDR or SRM to be supportive, opposed, or ambiguous.

Such summary assessments were provided by ascertaining whether the majority of experts, and specifically the content of the overall statements, identified a clear role or stance of a given actor group. Experts might mention a group because it had a clearly and self-evident supportive or oppositional role for CDR and/or SRM or in order to express that such an actor group was likely to become important in the future. If experts were split on a group being supportive or opposed, or if there was not a mention of an actor being overtly supportive or oppositional but rather that they generally would have a part to play, then this was classified as "ambiguous". In total, the most common status assigned was that of "ambiguous", broadly reflecting this latter kind of connection, albeit closely followed by "supportive"; much less common, however, were expert statements about actor groups being oppositional to CDR or SRM, with a majority of those handful of actor groups mentioned in such a sense relating to the opposition of various ENGO and civil society groups (e.g. Low et al., 2022b).

# Annex II: List of 125 semi-structured expert interview respondents

| **Name** | **Actor Type** | **Gender** | **Country** | **Institution** |
| --- | --- | --- | --- | --- |
| [Anonymous Aerospace Engineer] | Private Sector + Industrial Associations | Male | Germany | [Aerospace and space systems company focusing on integrated spacecraft] |
| Aganaba, Timiebi | Universities + Research Institutes | Female | USA | Arizona State University |
| Asayama, Shinichiro | Government + Intergovernmental Organizations | Male | Japan | National Institute for Environmental Studies |
| Bauer, Christopher Dean 'Casey' | Private Sector + Industrial Associations | Male | USA | Raytheon Space and Defense |
| Bazilian, Morgan | Universities + Research Institutes | Male | USA | Colorado School of Mines |
| Bellamy, Rob | Universities + Research Institutes | Male | United Kingdom | University of Manchester |
| Beuttler, Christoph | Private Sector + Industrial Associations | Male | Switzerland | Climeworks |
| Biermann, Frank | Universities + Research Institutes | Male | Netherlands | Utrecht University |
| Boettcher, Miranda | Universities + Research Institutes | Female | Germany | Institute for Advanced Sustainability Studies (IASS) |
| Brauer, Uwe | Private Sector + Industrial Associations | Male | Germany | Planetary Sunshade Foundation |
| Brickett, Lynn | Government + Intergovernmental Organizations | Female | United States | Department of Energy |
| Briggs, Chad | Universities + Research Institutes | Male | USA | University of Alaska, Anchorage |
| Brown, Marilyn | Universities + Research Institutes | Female | USA | Georgia Institute of Technology |
| Bruce, John | Private Sector + Industrial Associations | Male | Canada | Carbon Engineering |
| Buck, Holly Jean | Universities + Research Institutes | Female | USA | University at Buffalo |
| Burns, Wil | Universities + Research Institutes | Male | USA | American University |
| Caldeira, Ken | Universities + Research Institutes | Male | USA | Breakthrough Energy, Carnegie Institution for Sciences, and Stanford University, and Stanford University |
| Camilloni, Ines | Universities + Research Institutes | Female | Argentina | University of Buenos Aires (and Harvard University) |
| Carton, Wim | Universities + Research Institutes | Male | Sweden | Lund University |
| Centers, Ross | Private Sector + Industrial Associations | Male | Germany | Planetary Sunshades |
| Chalecki, Beth | Universities + Research Institutes | Female | USA | University of Nebraska Omaha |
| Chavez, Anthony E. | Universities + Research Institutes | Male | USA | Northern Kentucky University |
| Clarke, Leon | Universities + Research Institutes | Male | USA | University of Maryland |
| Clarke, William S. (Sev) | Private Sector + Industrial Associations | Male | Australia | Winwick Business Solutions |
| Cobo Gutiérrez, Selene | Universities + Research Institutes | Female | Switzerland | ETH Zurich |
| Cox, Emily | Universities + Research Institutes | Female | United Kingdom | Cardiff University |
| Creutzig, Felix | Universities + Research Institutes | Male | Germany | Mercator Research Institute on Global Commons and Climate Change (MCC) |
| Delina, Laurence | Universities + Research Institutes | Male | Hong Kong | Hong Kong University of Science and Technology |
| Di Marco, Leon | Private Sector + Industrial Associations | Male | United Kingdom | FSK Technology Research - Consultant |
| Dooley, Kate | Universities + Research Institutes | Female | Australia | University of Melbourne |
| Draper, Kathleen | Civil Society | Female | USA | International Biochar Initiative |
| Elliott, David | Universities + Research Institutes | Male | UK | The Open University |
| Erbay, Yorukcan | Private Sector + Industrial Associations | Male | United Kingdom | Element Energy |
| Felgenhauer, Tyler | Universities + Research Institutes | Male | USA | Duke University |
| Florin, Marie-Valentine | Universities + Research Institutes | Female | Switzerland | EPFL International Risk Governance Center (IRGC) |
| Forster, Piers | Universities + Research Institutes | Male | United Kingdom | University of Leeds |
| Frumhoff, Peter | Civil Society | Male | USA | Union of Concerned Scientists |
| Fuhrman, Jay | Government + Intergovernmental Organizations | Male | United States | Pacific Northwest National Laboratory (PNNL) |
| Fuss, Sabine | Universities + Research Institutes | Female | Germany | Mercator Research Institute on Global Commons and Climate Change (MCC) |
| Gambhir, Ajay | Universities + Research Institutes | Male | United Kingdom | Imperial College London |
| Geden, Oliver | Government + Intergovernmental Organizations | Male | Germany | German Institute for International and Security Affairs (SWP) |
| Ghosh, Arunabha | Civil Society | Male | India | Council on Energy, Environment and Water (CEEW) |
| Grant, Neil | Universities + Research Institutes | Male | United Kingdom | Imperial College London |
| Gruebler, Arnulf | Universities + Research Institutes | Male | Austria | International Institute for Applied Systems Analysis (IIASA) |
| Guillen Gosalbez, Gonzalo | Universities + Research Institutes | Male | Switzerland | ETH Zurich |
| Haberl, Helmut | Universities + Research Institutes | Male | Germany | BOKU Vienna |
| Haigh, Joanna | Universities + Research Institutes | Female | United Kingdom | Imperial College London / Grantham Institute |
| Hamilton, Clive | Universities + Research Institutes | Male | Australia | Charles Stewart University |
| Hartmann, Jens | Universities + Research Institutes | Male | Germany | University of Hamburg |
| Hawkes, Adam D. | Universities + Research Institutes | Male | United Kingdom | Imperial College London |
| Healey, Peter | Universities + Research Institutes | Male | United Kingdom | Oxford University |
| Heap, Richard | Civil Society | Male | United Kingdom | Carbon Removal Centre, Foresight Transitions |
| Hepburn, Cameron | Universities + Research Institutes | Male | United Kingdom | Oxford University |
| Herzog, Howard | Universities + Research Institutes | Male | United States | MIT |
| Heyen, Daniel | Universities + Research Institutes | Male | Germany | TU Kaiserslautern (formerly ETHZ) |
| Heyward, Clare | Universities + Research Institutes | Female | Norway | UiT - the Arctic University of Tromso |
| Honegger, Matthias | Universities + Research Institutes | Male | Germany | Institute for Advanced Sustainability Studies (IASS) |
| Horton, Joshua B. | Universities + Research Institutes | Male | USA | Harvard University |
| Irvine, Pete | Universities + Research Institutes | Male | United Kingdom | UCL |
| Jinnah, Sikina | Universities + Research Institutes | Female | USA | UC Santa Cruz |
| Johnson, Les | Government + Intergovernmental Organizations | Male | USA | NASA Marshall Space Flight Center |
| Kammen, Daniel | Universities + Research Institutes | Male | USA | UC Berkeley |
| Karami, Khalil | Universities + Research Institutes | Male | Slovenia/Germany | University of Ljubljana/University of Leipzig |
| Karlsberg Schaffer, Madeleine | Civil Society | Female | USA | SilverLining |
| Keller, David | Universities + Research Institutes | Male | Germany | GEOMAR - Helmholtz Centre for Ocean Research Kiel |
| Keller, Klaus | Universities + Research Institutes | Male | USA | Penn State University |
| Kravitz, Ben | Universities + Research Institutes | Male | USA | Indiana University |
| Kruger, Tim | Private Sector + Industrial Associations | Male | UK | Origen Power |
| Kuswanto, Heri | Universities + Research Institutes | Male | Indonesia | Institut Teknologi Sepuluh Nopember |
| Lawrence, Mark | Universities + Research Institutes | Male | Germany | Institute for Advanced Sustainability Studies (IASS) |
| Lehmann, Johannes | Universities + Research Institutes | Male | USA | Cornell University |
| Lenton, Andrew | Government + Intergovernmental Organizations | Male | Australia | CSIRO |
| Lin, Albert | Universities + Research Institutes | Male | USA | UC Davis |
| MacMartin, Doug | Universities + Research Institutes | Male | USA | Cornell University |
| Mahajan, Aseem | Universities + Research Institutes | Male | United States | Harvard University |
| Malik, Abdul | Universities + Research Institutes | Male | Saudi Arabia | King Abdullah University of Science and Technology (formerly Grantham Institute) |
| McLaren, Duncan | Universities + Research Institutes | Male | United Kingdom | Lancaster University |
| Mengis, Nadine | Universities + Research Institutes | Female | Germany | GEOMAR - Helmholtz Centre for Ocean Research Kiel |
| Merk, Christine | Universities + Research Institutes | Female | Germany | Kiel Institute for the World Economy |
| Michaelowa, Axel | Universities + Research Institutes / Private Sector + Industrial Associations | Male | Switzerland | University of Zurich / Perspectives Climate Group |
| Montserrat, Francesc | Universities + Research Institutes | Male | Netherlands | Project Vesta / Royal Boskalis Westminster N.V. |
| Moore, John | Universities + Research Institutes | Male | Finland | University of Lapland / Arctic Centre |
| Moreno-Cruz, Juan | Universities + Research Institutes | Male | Canada | University of Waterloo |
| Morrow, David | Universities + Research Institutes | Male | USA | American University |
| Muri, Helene | Universities + Research Institutes | Female | Norway | Norwegian University of Science and Technology (NTNU) |
| Obersteiner, Michael | Universities + Research Institutes | Male | United Kingdom | Oxford University |
| Odoulami, Romaric | Universities + Research Institutes | Male | South Africa | University of Cape Town |
| Parker, Andy | Civil Society | Male | UK | SRM Governance initiative |
| Parson, Edward 'Ted' A. | Universities + Research Institutes | Male | USA | UCLA |
| Pasztor, Janos | Civil Society | Male | Switzerland | Carnegie Climate Governance Initiative |
| Pidgeon, Nick | Universities + Research Institutes | Male | United Kingdom | Cardiff University |
| Pinto, Izidine | Universities + Research Institutes | Male | South Africa | University of Cape Town |
| Pongratz, Julia | Universities + Research Institutes | Female | Germany | University of Munich |
| Preston Aragonès, Mark | Civil Society | Male | Norway | Bellona Foundation |
| Rahman, Mohammed Mofizur | Universities + Research Institutes | Male | Germany | TH Cologne - University of Applied Sciences |
| Raimi, Kaitlin T. | Universities + Research Institutes | Female | United States | University Michigan |
| Reiner, David | Universities + Research Institutes | Male | United Kingdom | Cambridge University |
| Renforth, Phil | Universities + Research Institutes | Male | United Kingdom | Heriot-Watt University |
| Reynolds, Jesse | Universities + Research Institutes | Male | USA/Netherlands | UCLA/Independent Consultant |
| Rickels, Wilfried | Universities + Research Institutes | Male | Germany | Kiel Institute |
| Robock, Alan | Universities + Research Institutes | Male | USA | Rutgers University |
| Rothman, Dale | Universities + Research Institutes | Male | USA | University of Denver |
| Rouse, Paul | Universities + Research Institutes | Male | United Kingdom | University of Southampton |
| Schleussner, Carl | Civil Society | Male | USA | Climate Analytics |
| Schmidt, Joern | Universities + Research Institutes | Male | Germany | Kiel Institute |
| Schneider, Linda | Civil Society | Female | Germany | Heinrich Boell Foundation |
| Scott, Vivian | Universities + Research Institutes | Male | United Kingdom | Edinburgh University |
| Simonelli, Lucia | Civil Society | Female | United States | Carbon 180 |
| Smith, Pete | Universities + Research Institutes | Male | United Kingdom | University of Aberdeen |
| Smith, Steve | Universities + Research Institutes | Male | United Kingdom | Oxford University |
| Smith, Wake | Universities + Research Institutes | Male | USA | Harvard University |
| Spangenberg, Joachim | Universities + Research Institutes | Male | Germany | Sustainable Europe Research Institute SERI Germany e.V |
| Stephens, Jennie | Universities + Research Institutes | Female | USA | Northeastern University |
| Stoefs, Wijnand | Civil Society | Male | Belgium | Carbon Market Watch |
| Sugiyama, Masahiro | Universities + Research Institutes | Male | Japan | University Tokyo |
| Sunny, Nixon | Universities + Research Institutes | Male | United Kingdom | Imperial College London |
| Surprise, Kevin | Universities + Research Institutes | Male | USA | Mount Holyoke College |
| van Vuuren, Detlef | Government + Intergovernmental Organizations | Male | Netherlands | PBL Netherlands Environmental Assessment Agency |
| Vaughan, Nem | Universities + Research Institutes | Female | United Kingdom | University of East Anglia |
| Victor, David | Universities + Research Institutes | Male | USA | UC San Diego |
| Vivian, Chris | Government + Intergovernmental Organizations | Male | UK | GESAMP |
| Wagner, Gernot | Universities + Research Institutes | Male | USA | NYU |
| Wolske, Kimberly S. | Universities + Research Institutes | Female | United States | University Chicago |
| Wood, Robert | Universities + Research Institutes | Male | USA | University of Washington |
| Workman, Mark | Universities + Research Institutes | Male | UK | Energy Futures Lab, Imperial College London |

Note: All interviewee respondents gave oral permission to participate in our study during each interview, followed by written permission via a follow-up email. All but one of our experts (“Anonymous Aerospace Engineer”) consented for their names to be listed in this Annex.

# Annex III: Full GENIE semi-structured expert interview question and sub-question sets

| **Topic** | **Central question** | **Sub-questions** |
| --- | --- | --- |
| **1. Innovation** | Which particular options have high or low innovation potential in technical, communication, societal appraisal, and policy dimensions? | What are critical innovation gaps?  Which particular options have high or low learning potential?  What is the potential for massive scale up or scaling? |
| **2. Coupling** | What energy systems or other sociotechnical systems could or should be coupled to carbon removal? | What are complementary (or alternate) technologies and/or upstream and downstream technologies (or systems) a la the Technology Environment Analysis?  In what ways might greenhouse gas removal or solar radiation management tradeoff with, or further strengthen, renewable energy deployment? |
| **3. Business models** | What business models and markets could carbon removal create or disrupt? | Do you see the technologies benefitting new entrants, or incumbents?  Who is financing development or deployment?  What policies or changes in governance are needed? |
| **4. Risks** | Which serious risks (e.g., social, political, military, ethical, environmental) may arise? | What are possible risks for climate mitigation or adaptation?  What about the risk of accidents?  Is there a risk of weaponization? |
| **5. Sustainability** | What are the synergies and trade-offs of deployment for the Sustainable Development Goals and other societal objectives? | On balance are there more synergies or trade-offs?  Which are the most important? |
| **6. Justice** | What vulnerable groups could be affected, positively or negatively? | Who is most vulnerable?  What about impacts on indigenous groups?  What about future generations? |
| **7. Actors** | Who are the relevant (or most important) actors (or stakeholders / networks), e.g. for commercialization, development, and/or acceptability? | Who are the most powerful actors?  Are there any missing or invisible actors?  What about the military, or other non-traditional or subnational actor networks? |

Source: Authors.
